# 3D comparative evaluation and correlation of condylar morphology in bruxers and non-bruxers

**DOI:** 10.12688/f1000research.133773.1

**Published:** 2023-09-01

**Authors:** Aakanksha Tiwari, Suwarna Dangore-Khasbage

**Affiliations:** 1 Department of Oral Medicine and Radiology, Datta Meghe Institute of Higher Education and Research, Wardha, Maharashtra, 442001, India; 2 Department of Oral Medicine and Radiology, Datta Meghe Institute of Higher Education and Research, Wardha, Maharashtra, 442001, India

**Keywords:** Temporomandibular joint, TMJ, Bruxers, CBCT, Condyle, Condylar morphology

## Abstract

Parafunctional habits like bruxism may have a deleterious effect on the temporomandibular joint. It can lead to the alteration of condylar morphology which can further lead to temporomandibular joint disorders. The early three-dimensional evaluation may help detect morphological changes in the condyle and reduce the risk of developing temporomandibular disorders. It will also help to make patients aware of the condition and encourage prompt treatment of parafunctional habits. To do a 3-Dimensional comparative evaluation and correlation of condylar morphology in bruxers and non-bruxers. 90 patients with parafunctional habits will be evaluated on cone beam computed tomography in two groups (control group, study group) of 45 each, for alteration in condylar morphology based on the suggested classification. Results will be formulated based on the classification of condylar morphology. Evaluation of condylar morphology will be done on cone beam computed Tomography based on the amount of variation in condylar morphology in bruxers as compared to non-bruxers. Thorough clinical examination and early evaluation of condylar morphology on cone beam computed tomography will help early detection of changes in the morphology of condyle and prompt treatment to reduce the risk of development of temporomandibular disorders.

## Introduction

The Temporomandibular joint (TMJ) is a unique joint in our body that allows movement of the mandible in different direction. ^1^ The functional balance of the TMJ is maintained by mastication pattern *i.e.* the ideal bilateral mastication pattern is important in maintaining the functionality of TMJ. ^1^ Different forces that are exerted on the craniomandibular joint have an effect on the morphology of the bony components. This may lead to changes in the shape and thickness of these components. ^1^

One of the important bony components to the TMJ is the mandibular condyle. The condyle articulates with the glenoid fossa and brings about the movement of mandible. ^2^ The shape and morphology of condyle may be altered due to various reasons. One common reason being tooth loss. ^2^ Tooth loss, due to any reason, causes inappropriate distribution of masticatory forces leading to structural alterations in condyle. ^2^

Clinical examination alone is not satisfactory to evaluate structural changes in the condyle. ^1^ Due to the complex anatomy of maxillofacial region, adjacent structures may be superimposed, and it becomes tough to carry out accurate radiographic examination of the TMJ. Numerous radiographic techniques are being used to evaluate the TMJ like orthopantomograms, reverse Townes, lateral oblique, transcranial, transorbital, transpharyngeal *etc.* ^2^ These radiographic images are two dimensional and show superimposition of surrounding structures. Cone beam computed tomography (CBCT) has been indicated as an accurate and efficient imaging technique for determining structures in maxillofacial region by various studies performed previously. ^3^

CBCT is a three dimensional imaging technique which gives accurate details of the structure without superimposition and deformity. ^2^ Clinical examination of the TMJ can lead to the diagnosis of TMJ disorders, but it is recommendable to use clinical and radiographic evaluation in combination to reach an accurate diagnosis. ^1^ A number of studies have been done to evaluate the morphological changes in condyle due to loss of teeth. Eda Didem Yalcin *et al.* ^4^ in 2019 conducted a study to evaluate condylar morphology in relation with age, sex and edentulism using CBCT. Another study was performed by Dr. Saraswathi Gopal *et al.* ^5^ which showed that evaluation of condylar morphology helps in detection of the temporomandibular joint disorders in early stages. But on searching existing literature, there was a lack of studies which depict comparative changes in condylar morphology in bruxers and non-bruxers with the use of CBCT. Taking into account the lack of enough studies on condylar morphological changes with CBCT imaging in bruxers and non-bruxers, the purpose of this study is to correlate the morphological changes of condyle in bruxers and non-bruxers using CBCT.

## Aim and objectives

### Aim

Dimensional comparative evaluation and correlation of condylar morphology in bruxers and non-bruxers

### Objectives

• 3D evaluation and correlation of condylar morphology in non-bruxers
• 3D evaluation and correlation of condylar morphology in bruxers
• Comparison and correlation of condylar morphology in Bruxers and non-bruxers using CBCT

**Trial design**

• Clinical Trial, Comparative, Parallel group study. ^6^

## Methods

### Participants, intervention and outcome

*Study setting*

Academic Hospital, Sharad Pawar Dental College and Hospital, Sawangi Meghe, Wardha 442001

The study will be performed in India.

The study has been approved by “INSTITUTIONAL ETHICS COMMITTEE (IEC)” of Datta Meghe Institute of Higher Education And Research (Deemed to be University), Sawangi (Meghe), Wardha. This prospective study will be conducted in Oral Medicine and Radiology Department at Sharad Pawar Dental College.

### Inclusion criteria

Patient between age group of 40-60 years will be included in the study. Those patients with a complete set of dentition (except third molars) and give history of bruxism for a duration of more than a year, and complaining of orofacial pain, which on clinical examination will reveal severe attrition leading to reduced vertical height will be included in the study group. The control group will be formed by the patients with complete set of dentition (except third molar) with no complaint of orofacial pain, no history of bruxism, no evidence of attrition or reduced vertical height will be included in control group.

These criteria will be evaluated by the post graduate student performing the study and will be confirmed by the senior master of dental surgery (MDS) staff in the Department of Oral Medicine and Radiology.

### Exclusion criteria

Patients who will give history of surgery, fracture, congenital anomalies of TMJ, history of trauma from occlusion, any systemic disease affecting the bones, history of consumption of medications affecting the bone, presence of pathologic lesions of jaws, and patients giving history of adverse habits like betel nut or kharra chewing and patients with developmental tooth anomalies will be excluded from the study.

Eligibility criteria for Center will be availability of CBCT machine, Planmeca Romexis viewer will be used for analysis of the three-dimensional reconstructed images.

The intervention will be performed by the Post Graduate student under the supervision of radiologist.

### Interventions

The patient reporting to the Department of Oral Medicine and Radiology, Sharad Pawar Dental College and Hospital, Sawangi Meghe, Wardha, 442001 and willing to participate in the study will be taken for the study.

Detailed history and thorough clinical examination of each patient will be performed after taking informed written consent from each patient. Detailed history of the patient will be used to rule out systemic disease affecting joints, history of medications affecting the joint anatomy, history of trauma to the temporomandibular joint or surgical intervention for temporomandibular joint.

Examination of the TMJ will be done to rule out any TMJ disorder. This will be performed by Post Graduate Student (the author) and will be confirmed by MDS faculty. Inspection of joint movement on opening and closing of mouth (restricted mouth opening, pain on opening jaw, deviation or deflection while opening jaw).

This will be performed by Post Graduate Student (the author). The patient will be asked to slowly open and close the mouth. The movements on the mandible will be closely inspected for any restriction in opening mouth, pain and tenderness while opening the mouth will be inferred by the patient’s facial expressions, any deviation or deflection in the path of closure will be inspected closely. Palpation of joint will be used to evaluate any clicking sound, crepitus, and tenderness while opening and closing of jaws. Palpation of the TMJ will be done by post graduate student and will be cross checked by MDS faculty. Patient will be positioned upright in the dental chair. Palpation of TMJ will be done by extra auricular or intra auricular methods. Palpation will be done by standing on 10 or 11’o clock position *i.e.* in front of the patient. Intra auricular palpation will be done using little finger. By placing little finger in both the ears, the posterior pole of condylar head can be palpated with pulp of little finger, during mandibular movements. Extra auricular palpation is done by placing index finger in pre-auricular region about 1.5 cm medial to tragus of ear. The lateral pole of condyle is accessible during this examination.

*Examination of muscles of mastication*

Palpation of masseter, temporalis, lateral and medial pterygoid muscles will be done for any tenderness if present. Thorough intraoral examination will be done to evaluate the patient with reduced vertical height due to bruxism. Clinical examination will be done by the post graduate student *via* thorough intra-oral inspection by correlating with the Tooth Wear Index. The findings will be confirmed by the Master of Dental Surgery faculty. Temporalis and masseter muscle will be palpated through entire length and breadth with clenched teeth with help of index and middle finger from 12’o clock position. Lateral pterygoid muscle will be palpated by placing index finger or little finger lateral to maxillary tuberosity and medial to coronoid process. Medial pterygoid muscle will be palpated by placing finger at a 45° angle in floor of mouth near base of relaxed tongue. The opposite hand will be used to extra orally to palpate posterior and inferior portion of insertion. The body of muscle will be palpated by rotating index finger upwards against the muscle to near its origin on tuberosity.

**Figure 1.**
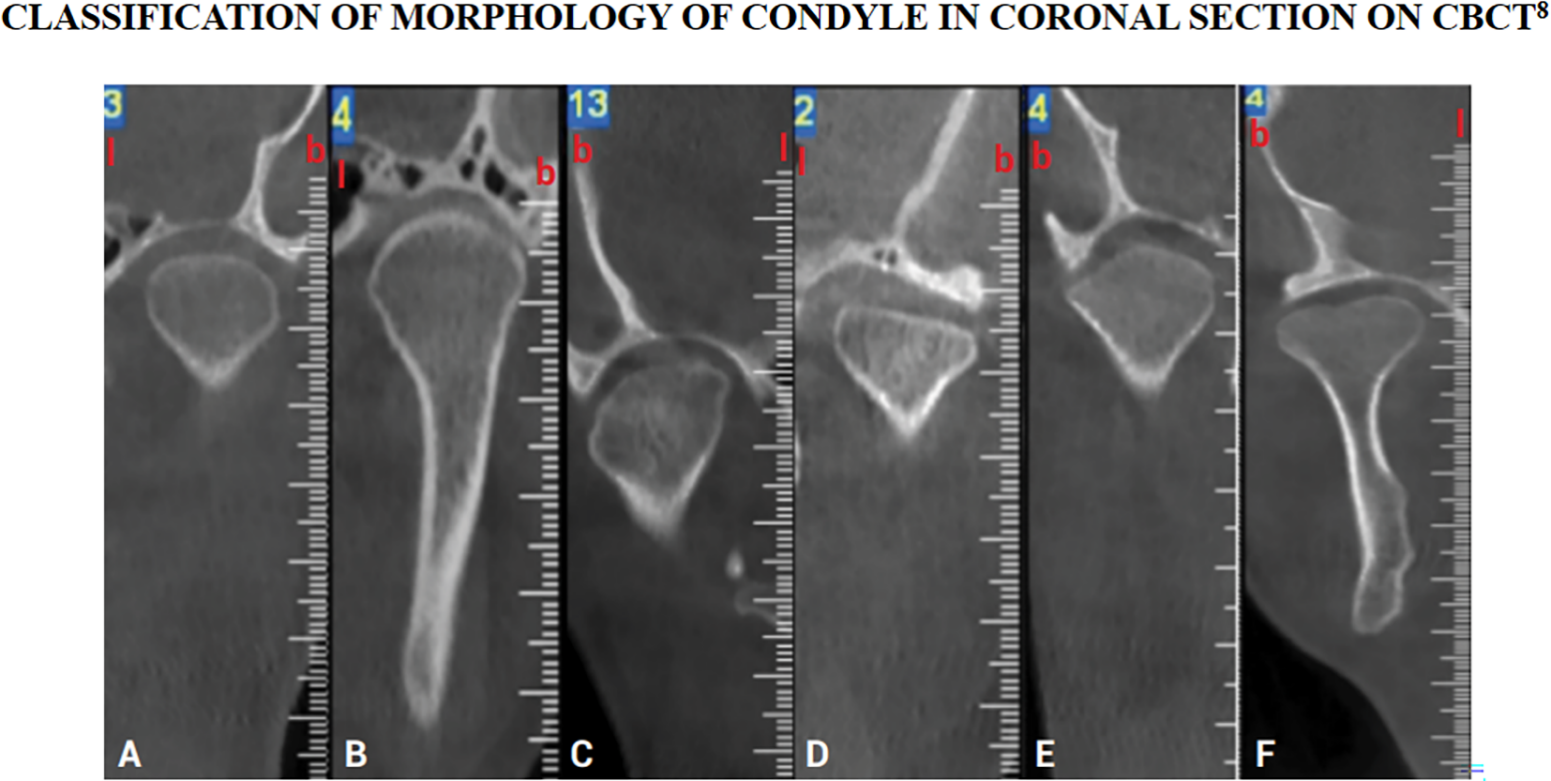
Classification of condylar morphology on coronal section of temporomandibular joint on cone beam computed tomography. The figure is available under the CC-BY-4.0 license from Ref. 7.

**Figure 2.**
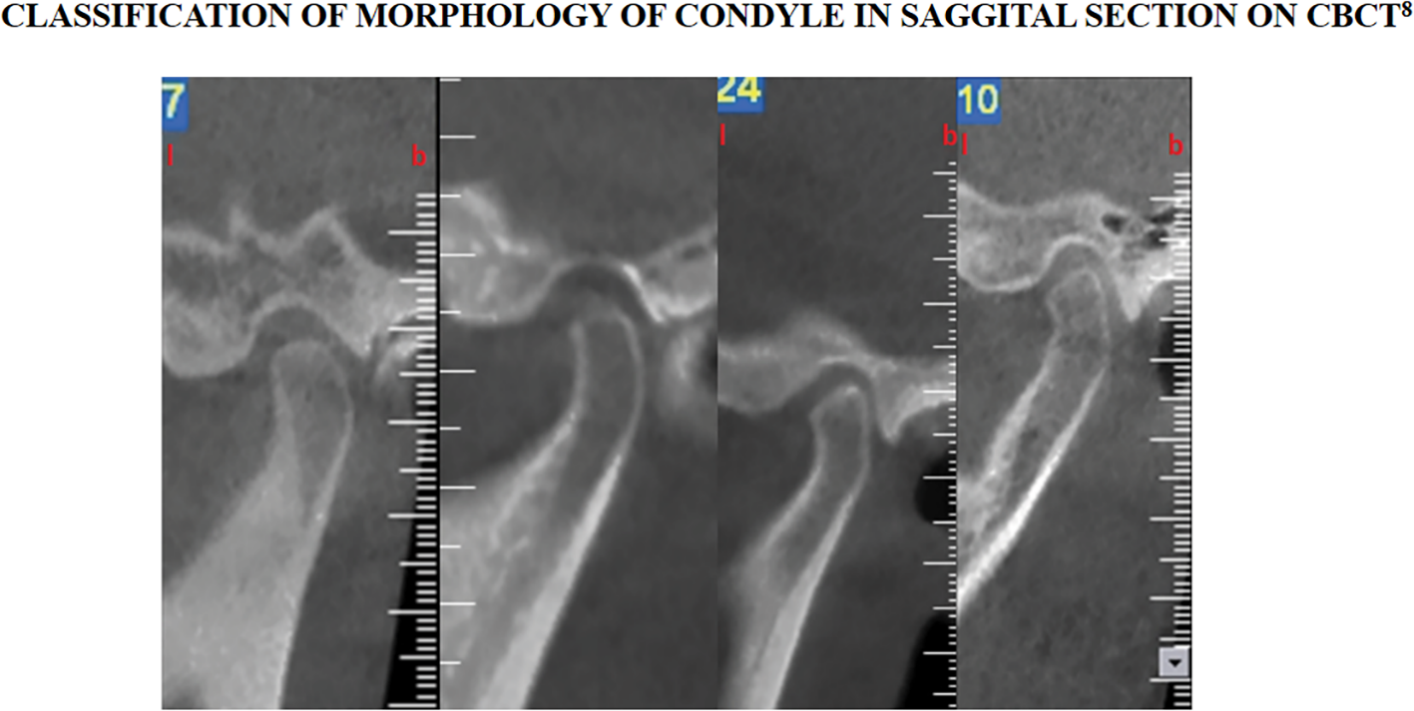
Classification of condylar morphology on sagittal section of temporomandibular joint on cone beam computed tomography. The figure is available under the CC-BY-4.0 license from Ref. 7.

**Figure 3.**
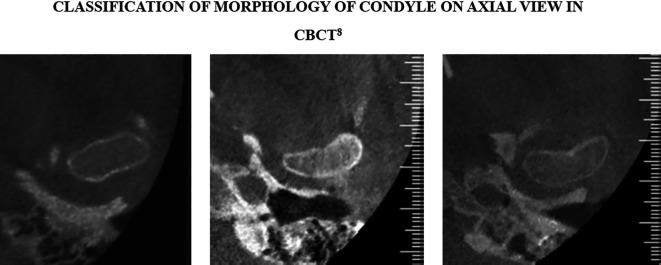
Classification of condylar morphology on axial section of temporomandibular joint in cone beam computed tomography. The figure is available under the CC-BY-4.0 license from Ref. 7.

Intervention will be performed using the 3-dimensional x-radiation technique, CBCT. Evaluation of condylar morphology will be done in coronal, sagittal and axial planes as suggested in figures below ( Figures 1– 3).

*Modification*

In case of over radiation exposure, if patient is already subjected to radiation previously for multiple times as in conditions where patients have been subjected to radiographic evaluation multiple times or in condition where patients have been taking radiotherapy, there is risk of worsening the disease due to radiation exposure the patient will be excluded from the study. A patient will also be excluded if they don’t give consent for the radiation exposure

*Adherence*

Intervention will be monitored ( *i.e.* X radiation in the form of CBCT exposure, its exposure parameters monitoring) by the post graduate student and the radiologist for adherence.

### Outcomes

The primary outcome expected is that there will be change in condylar morphology *i.e.* flattening, osteophyte, sclerosis, surface erosion in patients with habit of bruxism. We also expect that the use of CBCT could be beneficial for making patients aware of the condition and need prompt intervention at that particular time so that the condition does not progress to temporo-mandibular disorders. The intervention will be done after the confirmation of habit of bruxism for duration of at not less than 1 year.

### Participant timeline

The patients after reporting to the Department of Oral Medicine and Radiology will be screened to meet the eligibility criteria to be included in the study by thorough clinical examination during first visit and patient will be allocated in study and control group in the same visit based on the findings of clinical examination. Informed written consent will be taken from the patient. After getting the consent, patient will be subjected to CBCT exposure for evaluation. Assessment will be done and variables will be observed and evaluated by the post graduate student and masters of dental surgery faculty (as seen in Table 6).

### Sample size

$$P=\frac{P_{1}+{\mathrm{rP}}_{2}}{r+1}$$

$$n\geq \frac{{\left[Z_{1-\propto /2},\sqrt{\left(r+1\right)p\left(1-p\right)},+,Z_{1-\beta },\sqrt{{\mathrm{rp}}_{1}\left(1-p_{1}\right)+p_{2}\left(1-p_{2}\right)}\right]}^{2}}{r{\left(p2-p1\right)}^{2}}$$

Alpha (α): 0.05

Beta (β): 0.2

Proportion in group 1: 0.758

Proportion in group 2: 0.458

Proportion of condylar morphological change *i.e.* proportion of surface erosion in group 1 (control group: non-bruxers) = 75.8% (% of surface erosion of mandibular condyle) and group 2 (study group: bruxers) = 48.5% (% of surface erosion of mandibular condyle).

### Assumption

$$\text{Type}\;I\;\text{error}=5\%=Z_{1-\frac{\alpha }{2}}=1.96$$

(1-β) Power of the test = 100-20 = 80%, considering 20% as Type II error = 0.84

r = Ratio = 1:1

Total sample size = 49 each group.

Study reference: Ref. 1.

### Recruitment

Each and every patient reporting in the OPD in Oral Medicine and Radiology department will be examined for habit of bruxism. We will also have some collaboration with the Department of Orthodontics for patients with bruxism and they will recommend patients for us.

### Assignment of interventions

*Allocation: sequence generation*

Computer generated random numbers method will be used for generation of the sequence of the study participants. The list of the study participants will be fed as raw data in the computer and it will be randomly shuffled. The numbers allotted to the participants will be computer generated random numbers and participants will be selected randomly and the arranged in sequence as needed.

*Allocation concealment mechanism*

Allocation concealment mechanism will be sequentially numbered.

### Blinding

Blinding will be done while evaluating the CBCT images of group 1 and group 2 subjects by post graduate student and two radiologists (who will be blinded).

### Data collection, management, and analysis

*Data collection plan*

The patients reporting to the Department of Oral Medicine and Radiology, will be evaluated for habit of bruxism using a questionnaire. ^8^^,^^9^

After diagnosing the patients, written consent will be given by them and the procedure of intervention will be explained thoroughly.

Following collection of consent the bruxers and non-bruxers will be evaluated prospectively using CBCT. This will form two separate groups as follows:

- Bruxers: 49 patients (based on the questionnaire given to the patient and thorough clinical examination)
- Non-Bruxers: 49 patients (control group) (based on the questionnaire given to the patient and thorough clinical examination)

No blinding will be applied while selecting the groups. Evaluation of condylar morphology will be done based on the following classifications:

A) Classification of Condylar Morphology on Coronal section of TMJ on CBCT ^10^
B) Classification of Condylar Morphology on Sagittal section of TMJ on CBCT ^10^
C) Classification of condylar Morphology on Axial section of TMJ in CBCT ^7^

The patients diagnosed with bruxism based on the questionnaire will be thoroughly examined clinically for the wearing of teeth, using a “Tooth Wear Index”. The tooth wear index distributed from score 0-4 will be used. ^9^^,^^11^

After diagnosing the patients, written consent will be given by them and the procedure of intervention will be explained thoroughly.

### Data collection plan

*Retention*

The participation of the patients in the study will be increased by proper counselling regarding the condition. As the patients will not be aware of the situation because of no signs and symptoms of the disease, proper guidance and counselling will be helpful in making them aware of the consequences of the same.

*Data management*

The data of CBCT images of the bruxers and non-bruxers will be entered in a mastersheet with percentage of changes in condylar morphology in both the groups ( Tables 1– 4).

**Table 1.** Condylar morphological changes on coronal section of cone beam computed tomography (CBCT) in bruxers and non-bruxers.

Condylar morphological changes on coronal section of CBCT	Group 1 (Bruxers)	Group 2 (Non-Bruxers)	P-Value
Convex			
Round			
Flat			
Angled			

**Table 2.** Condylar Morphological Changes on sagittal section of cone beam computed tomography (CBCT) in bruxers and non-bruxers.

Condylar morphological changes on sagittal section of CBCT	Group 1 (Bruxers)	Group 2 (Non-Bruxers)	P-Value
Normal			
Flattening			
Erosion			
Deformity (beak shaped)			

**Table 3.** Condylar Morphological Changes on axial section of cone beam computed tomography (CBCT) in bruxers and non-bruxers.

Condylar morphological changes on axial section of CBCT	Group 1 (Bruxers)	Group 2 (Non-Bruxers)	P-Value
Oval			
Bean-shaped			
Conical			

**Table 4.** Evaluation of group 1 *i.e.* Bruxers using Tooth Wear Index.

Tooth Wear Index Score	Group 1 (Bruxers)	P Value
**0**		
**1**		
**2**		
**3**		
**4**		

### Statistical analysis

All statistical analysis will be completed using r software version 4.3 (RRID:SCR_001905).

Descriptive statistics for the qualitative variable will be reported in count and percentage.

*Quantitative analysis*

Quantitative analysis over continuous data (defect in the tooth in ***mm*** due to loss of enamel, exposure of dentin) will be reported in mean, standard deviation, median, maximum and minimum.

Unpaired t test or alternative Wilcoxon test will be used to find out significant difference in condylar morphology between group 1 (Bruxers) and group 2 (non-Bruxers).

Data for the Quantitative outcome variables *i.e.* the defect in teeth in mm due to loss of enamel, exposure of dentin, will be tested for the normality using Kolmogorov Smirnov test.

Non-normal data will be transformed into normal data by using mathematical algorithms. If data persists with non-normal distribution, then alternative test (Wilkoxon) will be used for Unpaired t test.

Wearing of teeth will be quantitatively measured using Tooth Wear Index Table 5. ^12^

**Table 5.** Evaluation of group 2 *i.e.* Non-Bruxers using Tooth Wear Index.

Tooth Wear Index Score	Group 2 (Non-Bruxers)	P Value
**0**		
**1**		
**2**		
**3**		
**4**		

**Table 6.** Participant timeline.

	Study period							
	Enrolment	Allocation	Post-allocation					Close-out
Timepoint**	*-t _1_*	0	*t _1_*	*t _2_*	*t _3_*	*t _4_*	*etc.*	*t _x_*
**Enrolment:**								
**Eligibility screen**	X							
**Informed consent**	X							
***[List other procedures]***	X							
**Allocation**		X						
**Interventions:**								
***[Intervention A- X rays using CBCT]***		X						
**Assessments:**								
***[Condylar morphology]***	X	X						
***[Flattening, osteophytes, sclerosis, surface erosion]***				X		X	etc.	X

*Quantitative variables:* Defect in the tooth in ***mm*** due to loss of enamel, exposure of dentin.

*Qualitative variables:* Condylar morphological changes in coronal, sagittal and axial sections on CBCT.

Inferential statistics will be performed on finding the significant difference among bruxers and non bruxers.

*Qualitative analysis*

The outcome variables flattening, osteophyte, sclerosis, surface erosion *i.e.* will be categorized for frequency distribution and will be tested for significant association among bruxers and non bruxers using the Chi square test.

The outcome variables flattening, osteophyte, sclerosis and surface erosion for tooth wear index distributed from score 0-4 will be categorized for frequency distribution and will be tested for significant association among bruxers and non bruxers using Chi square test.

### Monitoring

*Formal committee*

No specific data monitoring committee will be used for this study. The collected data will be monitored by the post graduate student, the MDS faculty and the radiologists. As this study will be performed by post graduate student only, there is no need of the specific committee for data monitoring.

*Interim analysis*

The data can be accessed by the post graduate student performing the study and by the MDS faculty who is monitoring the study. Final decision to terminate the trial will be with post graduate student and MDS faculty.

*Harms*

Patients will be closely monitored for any harmful effect of radiation after being exposed and if observed any, the intervention will be stopped immediately and patient will be excluded from the study.

*Auditing*

No such procedure is applied yet and is not planned.

## Ethics and dissemination

### Research ethics approval

The study was applied for ethical approval on **02/02/2023** and has been approved by the Institutional Ethics Committee of Sharad Pawar Dental College and Hospital, Wardha on **06/02/2023.**

- **Ethical approval number –** DMIHER (DU)/IEC/2023/567

### Protocol amendments

- Post graduate student and MDS faculty can be contacted for any modification in protocol

### Consent

- Consent will be taken by post graduate student

The written informed consent statement that will be used is available from Zenodo.

**DOI** - 10.5281/zenodo.7975639

**IMAGE URL** - https://zenodo.org/badge/DOI/10.5281/zenodo.7975639.svg

**TARGET URL -** https://doi.org/10.5281/zenodo.7975639

### Confidentiality

Details will shared only with the MDS faculty

*Dissemination*

This study protocol will be published in PubMed, Web of Science and Scopus Indexed Journal. When the study is completed it is expected that a manuscript detailing the results and all analysis will also be published in an indexed journal.

**Study status**

The study is not yet started. It is expected to be started by 12/06/2023 and expected to end before completion of MDS curriculum *i.e.* before October 2025.

## Discussion

### Key result

It is expected that there will be a significant alteration in condylar morphology in bruxers as compared to non-bruxers as observed on CBCT.

### Interpretation

Kazuhiro Yamada *et al.* (2001) ^2^ conducted a study to evaluate condylar morphology in subjects with self-reported parafunctional habits. This study included 94 female patients diagnosed with temporomandibular disorders based on the questionnaire and clinical examination. The results revealed condylar bony changes in 53 females out of 94 (56.4%). They concluded that subjects with bruxism and clenching showed significant condylar bony changes. Parafunctional habits revealed relationship condylar bony changes.

Ozlem Isnam *et al.* (2020) ^13^ conducted a study to evaluate the density, mineralization, and morphology of bone in patients with sleep bruxism. The study included 120 patients (60 bruxers and 60 non-bruxers) in which cortical width at different locations was evaluated using panoramic radiography. The results showed significant changes in bone density, morphology and mineralisation in bruxers. They concluded that there were defects in endosteal margins of cortex and cortical bone thickening in mental foramen region in bruxers.

Benedikt Sagl *et al.* (2022) ^14^ performed a study to evaluate the effect of grinding direction of teeth, morphological parameters of teeth, on TMJ. In Silico computer model of masticatory region of a male volunteer was made. The result of this study stated that bruxism increases load on TMJ. This study proposed an innovative, forward dynamic approach to study the effect of bruxism on TMJ loading.

Juan Zhang *et al.* (2022) ^15^ conducted a study to evaluate TMJ morphology in definite sleep bruxers using magnetic resonance imaging (MRI) and CBCT. 38 subjects were studied (19 definite sleep bruxers, 20 asymptomatic patients). The results showed higher prevalence of disc deformity and disc displacement and condylar bony changes in sleep bruxers.

Aghitsna Aulia Aufa *et al.* (2022) ^16^ conducted a cross sectional study to evaluate mandibular morphological changes in bruxers and non-bruxers using panoramic radiography. Samples of 30 patients were studied. The results were drawn based on the Levandoski’s basic reference lines. The conclusion stated that there was a significant relationship between mandibular morphological changes in bruxers.

### Generalisability

Early detection of alteration in condylar morphology in bruxers as observed on CBCT will help prompt treatment and will reduce the risk of developing temporomandibular disorders. As it is a 3D imaging technique, it will best reveal slightest of change in condylar morphology which will help in making the patient aware of the condition and offering accurate treatment.

## Data Availability

Zenodo: Tooth wear index, https://doi.org/10.5281/zenodo.7955094 ^12^ This project contains the following extended data:
- Tooth wear index.docx Zenodo: Questionnaire for bruxism, https://doi.org/10.5281/zenodo.7955064. ^9^ This project contains the following extended data:
- Questionnaire for bruxism.docx Zenodo: 3D comparative evaluation and correlation of condylar morphology in bruxers and non-bruxers, https://doi.org/10.5281/zenodo.7954711. ^6^
- SPIRIT_checklist.docx Data are available under the terms of the Creative Commons Attribution 4.0 International license (CC-BY 4.0).
