# Supplementary material for: 3D comparative evaluation and correlation of condylar morphology in bruxers and non-bruxers
Source: F1000Res. 2023 Sep 1;12:1099. [Version 1] doi: 10.12688/f1000research.133773.1 (PMC11391183; doi:10.12688/f1000research.133773.1)
Supplement: Peer review history

# Reviewer report: Reviewer response for version 1

**DOI:** 10.5256/f1000research.146786.r257842

**Published:** 2024-09-11

**Authors:** Dr. Garima Yeluri

**Affiliations:** 1 Teerthankar Mahavaeer Dental College & Research Center, UP, India

The Title of the study justifies the study design. Aims & Objectives are clearly mentioned. The study design is apt with inclusion and exclusion criterias along with the Statistical analysis design. The condylar morphology to be evaluated in in coronal, saggital and axial sections are completely explained in the methodology. The datasets designed are very clear. Overall the rationalle of the study is scientifically sound.

Is the study design appropriate for the research question?

Yes

Is the rationale for, and objectives of, the study clearly described?

Yes

Are sufficient details of the methods provided to allow replication by others?

Yes

Are the datasets clearly presented in a useable and accessible format?

No

Reviewer Expertise:

Temporomandibular joint disorders, Premalignant lesions and conditions, Orofacial pain

I confirm that I have read this submission and believe that I have an appropriate level of expertise to confirm that it is of an acceptable scientific standard.

# Reviewer report: Reviewer response for version 1

**DOI:** 10.5256/f1000research.146786.r286834

**Published:** 2024-06-25

**Authors:** Jing Guo

**Affiliations:** 1 Department of Orthodontics, Shandong University, Jinan, Shandong, China

This study used CBCT to compare the condylar morphological characteristics of patients with and without bruxism. The results not only confirmed the damage of the anatomical morphology of the temporomandibular joint in the progression of bruxism, but also provided some evidence for the understanding of its patho-physiological mechanism in the process of clinical diagnosis. In addition, the study also involved the comparison of tooth attrition index between the two groups, which provided clinical support for early intervention of bruxism to reduce dental tissue damages.

 The objectives of this study were clearly described.The formulation of scientific questions, the consistency of research subjects and the design of sample size were consistent with the methodology of clinical research.

Is the study design appropriate for the research question?

Yes

Is the rationale for, and objectives of, the study clearly described?

Yes

Are sufficient details of the methods provided to allow replication by others?

Yes

Are the datasets clearly presented in a useable and accessible format?

Yes

Reviewer Expertise:

1.Dental maxillofacial malformation associated with the airway physiology mechanism. 2.Associated mechanism of occlusion and temporomandibular joint function.

I confirm that I have read this submission and believe that I have an appropriate level of expertise to confirm that it is of an acceptable scientific standard.

# Response

**Published:** 2024-06-27

**Authors:** Aakanksha Tiwari

**Affiliations:** Oral Medicine and Radiology, Datta Meghe Institute of Higher Education and Research, Wardha, Maharashtra, India

Respected sir,

 I extend my heartfelt thanks to you for taking out your valuable time to review and approve my protocol and to provide your valuable views on my topic.

# Response

**Published:** 2024-07-04

**Authors:** Aakanksha Tiwari

**Affiliations:** Oral Medicine and Radiology, Datta Meghe Institute of Higher Education and Research, Wardha, Maharashtra, India

Thank you for your valuable reviews.
